# Cerebrovascular health impacts processing speed through anterior white matter alterations: a UK biobank study

**DOI:** 10.1038/s41598-025-93399-2

**Published:** 2025-03-21

**Authors:** Katie L. Moran, Craig J. Smith, Elizabeth McManus, Stuart M. Allan, Daniela Montaldi, Nils Muhlert

**Affiliations:** 1 Present Address: Division of Psychology, Communication and Human Neurosciences, School of Health Sciences, University of Manchester, Manchester, UK; 2 Geoffrey Jefferson Brain Research Centre, Manchester Academic Health Science Centre, Northern Care Alliance NHS Foundation Trust, University of Manchester, ManchesterManchester, UK; 3 Division of Cardiovascular Sciences, School of Medical Sciences, University of Manchester, Manchester, UK; 4 Manchester Centre for Clinical Neurosciences, Salford Royal Hospital, Northern Care Alliance NHS Foundation Trust, Salford, UK; 5 Division of Psychology and Mental Health, School of Health Sciences, University of Manchester, Manchester, UK; 6 Division of Neuroscience, School of Biological Sciences, University of Manchester, Manchester, UK

**Keywords:** White matter, Cerebrovascular, Processing speed, Cognition, Cerebrovascular disorders, Risk factors, Cognitive neuroscience

## Abstract

Cerebrovascular disease is associated with an increased likelihood of developing dementia. Cerebrovascular risk factors are modifiable and may reduce the risk of later-life cognitive dysfunction, however, the relationship between cerebrovascular risk factors, brain integrity and cognition remains poorly characterised. Using a UK Biobank sample of mid-to-old aged adults, without neurological disease, our structural equation mediation models showed that poor cerebrovascular health, indicated by the presence of cerebrovascular risk factors, was associated with slowed processing speed. This effect was best explained by anterior white matter microstructure (e.g. genu, anterior corona radiata), rather than posterior (e.g. splenium, posterior corona radiata)—the mediatory effect of anterior white matter strengthened further with age. Effects were also significantly reduced when considering other forms of cognition, demonstrating both regional- and cognitive-specificity. Our findings also illustrate that cerebrovascular risk factors cross-sectionally predict cognitive processing speed performance, which can be further strengthened by accounting for risk factor duration, particularly hypertensive duration. In summary, our study highlights the vulnerability of anterior regions and sensitivity of processing speed performance to cerebrovascular burden, and show this effect is amplified with age. We also highlight an improved method of cerebrovascular burden quantification, which accounts for factor duration, as well as risk factor presence and degree. Future work will aim to establish the role of medication and effective risk factor control in alleviating or preventing white matter pathology and cognitive dysfunction.

## Introduction

Cerebrovascular disease is closely linked with the development of later-life cognitive dysfunction and dementia^1,2^. Many of the risk factors associated with cerebrovascular disease are modifiable, such as hypertension and smoking status, which suggests they may be an optimal group of therapeutic targets with which to minimise or even prevent later cognitive dysfunction. However, in order to identify the risk factors which will yield maximal impact when targeted, a mechanistic understanding of the relationship between cerebrovascular health and cognition is needed; particularly during the earlier stages of this relationship, where intervention is more likely to have a beneficial impact on both brain integrity and cognitive function.

White matter is particularly vulnerable to vascular insult, due to its cellular composition and absence of collateral circulation^3^ and is likely key to understanding how poor cerebrovascular health influences cognition. Macroscale markers of white matter injury, such as white matter hyperintensities (WMHs) are consistently linked to both poor vascular health and cognitive dysfunction^4^. However, WMHs reflect advanced stages of both white matter injury and cerebrovascular disease, whereby it is unlikely that intervention at this stage would have a beneficial impact on brain health or cognitive function^5^. Diffusion imaging allows for microscale white matter changes to be mapped, which reflect a much earlier stage of injury that may be more receptive to intervention, relative to their macroscale counterparts^6^. These microscale measures of white matter integrity (WMI) may be particularly valuable when applied to studying middle-age populations where damage derived from poor cerebrovascular health is likely more subtle and difficult to detect. WMI is shown to correlate well with cerebrovascular health and individual risk factors, such as hypertension^7^, diabetes^8,9^ and obesity^10^, and is sensitive in predicting stroke risk^11^. Therefore, it’s likely that subtle WMI changes may act as a sensitive biomarker of early cerebrovascular burden. *Cerebrovascular burden* is characterised as the cumulation of risk factors, which are recognised to place functional on the vascular system, such as; hypertension, diabetes, high cholesterol, active smoking status, high BMI and/or high waist to hip ratio. In this study, individuals with high levels of cerebrovascular burden are not sufficiently advanced to have a cerebrovascular disease diagnosis but are at a greater risk of developing it.

A growing body of evidence suggests the impact of cerebrovascular burden on white matter may be region-specific. More specifically, the differences in the vascular characteristics of anterior and posterior white matter regions may moderate the impact of cerebrovascular burden on the brain, particularly in its early stages. For example, hypertension is associated with greater change in anterior cerebrovascular function, such as reduced cerebral blood flow, relative to posterior regions^12,13^. This suggests a unique vulnerability of anterior regions to impaired vascular health. Region-specific white matter damage may also explain some of the inconsistent findings in the literature. For example, one study found no correlation between superior longitudinal fasciculus (SLF) WMI and cerebrovascular burden^14^. However, using the same UK Biobank sample, albeit with small differences in exclusion criteria, another study found a strong relationship between global WMI and cerebrovascular burden^15^. In this study, global WMI was quantified as a latent construct using the following tracts - acoustic radiation, anterior thalamic, cingulum gyrus, and parahippocampal, corticospinal, forceps major and minor, inferior fronto-occipital, inferior longitudinal, middle cerebellar peduncle, medial lemniscus, posterior thalamic, superior longitudinal, superior thalamic, uncinate. The latter study also evidenced some anterior-specific effects of cerebrovascular burden, which were likely not sufficiently captured by the SLF alone in the former study. In addition, the former study used an aggregate method to quantify cerebrovascular burden, which may inflate the effects of noise and underestimated effects^14,16^. A large sample study investigating regionally specific effects of cerebrovascular burden, which adopts sufficiently sensitive metrics and quantification methods is necessary to address these shortfalls.

Given the proposed regional-specific impact of cerebrovascular burden, it follows that there may be some specificity to the cognitive consequences of this burden. Cerebrovascular disease has been linked with deficits in the cognitive processes supported by frontal regions, including processing speed, executive function and working memory^17–20^. However, processing speed is most consistently linked to cerebrovascular health and anterior white matter change^21^. Salthouse proposed that slowed processing speed precedes decline in other cognitive domains^22^. In support of this, previous work has shown that processing speed mediates the impact of vascular burden on other cognitive domains in a cohort of older adults^23^. Processing speed may therefore be particularly well suited as a candidate early marker of cerebrovascular burden. This is further supported by evidence of a strong link between WMI and slowed processing speed^21^. We can therefore postulate a pathway through which cerebrovascular burden alters white matter, and manifests behaviourally as subtle, but accumulating, cognitive change, likely beginning with alterations in speed of processing.

The *duration* of cerebrovascular risk factors may also significantly impact upon brain integrity and cognition. Studies reporting links between cerebrovascular burden, cognition and brain health traditionally quantify cerebrovascular burden based on the summed presence of known risk factors at the time of assessment^14,15^. However, this static approach may underestimate cerebrovascular burden, and consequently, its impact. Using this approach, an individual with a chronic history of hypertension would be considered to yield the same degree of burden as an individual with a recent diagnosis. This is inherently problematic, as the duration of hypertension is significantly exacerbates both white matter alterations^24^ and cognitive dysfunction^25^, and in particular, processing speed^26^. Additionally, smoking and diabetes duration are also thought to contribute to white matter change over time, but the cognitive impact of risk factor duration is not fully understood^9,27^.

While the evidence to date suggests a viable pathway through which early cerebrovascular burden may lead to cognitive impairment, further research is needed to characterise and better understand the specificity of this pathway. This will improve our understanding of cerebrovascular cognitive health from a systems perspective and will inform the future development of appropriate and sensitive cognitive measures, used to assess cognitive health in those with cerebrovascular burden. With this ambition in mind, the aims of the present study were twofold. First, we aimed to explore the relationship between cerebrovascular burden, WMI and cognition in mid-to-old aged participants and investigate whether any effects of cerebrovascular burden were specific to anterior WMI or to processing speed. We predicted that as cerebrovascular burden increases, processing speed will slow and that this effect will be mediated by anterior WMI. Furthermore, we also predicted that these effects would be specific to, or at least more pronounced for, processing speed, compared to other domains of cognition and to anterior WMI, rather than posterior WMI. We were also interested in whether the mediatory effect of anterior white matter integrity between cerebrovascular health and processing speed differs between mid-age and old-age groups. We repeated the analysis and compared the two groups to examine any differences, where we predicted that the effects are likely more modest in the mid-age group, in comparison to the old-age group across all paths. Our second aim was to assess whether cerebrovascular models which predict cognitive processing speed can be improved by accounting for risk factor duration. In this case, we estimated that models which accounted for the presence and duration of cerebrovascular risk factors will outperform static models of cerebrovascular burden in predicting processing speed performance.

## Results

### Demographic information, descriptive statistics & factor loadings

**Fig. 1:**
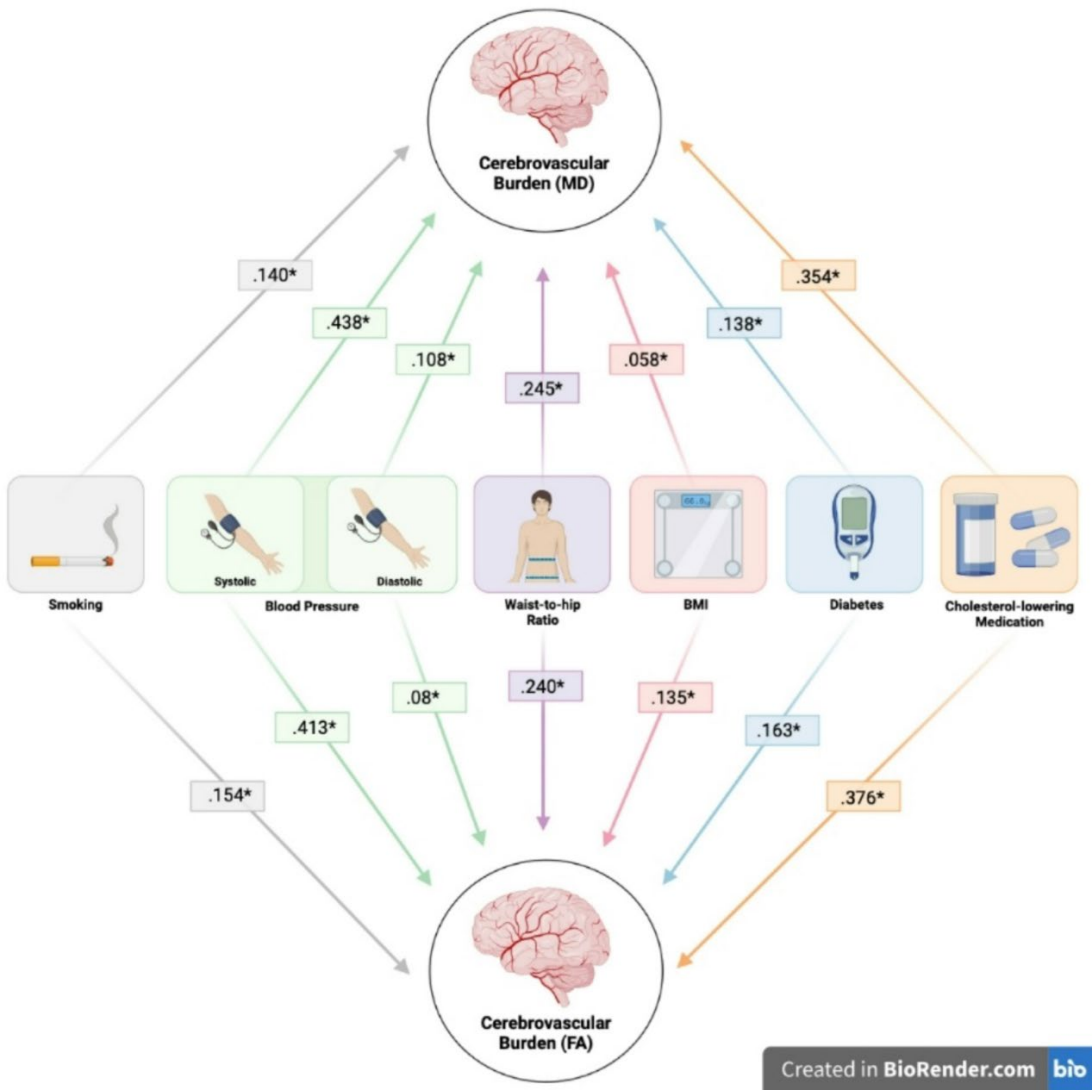
Factor loadings for each cerebrovascular risk factor onto the latent construct of ‘Cerebrovascular Burden’ in separate Fractional Anisotropy (FA) and Mean Diffusivity (MD) models. Each risk factor loaded significantly (*p < .001). This figure was created using BioRender.com.

Cerebrovascular, neuroimaging and cognitive data were analysed in a sample of 37,265, aged 40–75, derived from the UK Biobank. Descriptive statistics for demographic information and cerebrovascular risk factors are described in Table 1. Data shown are described prior to being transformed (e.g. BMI - log transformed) and scaled (all data). All variables loaded significantly (*p* < .001) onto their respective latent construct, which are depicted in Fig. 1; Tables 2 and 3, respectively.

**Table 1:**
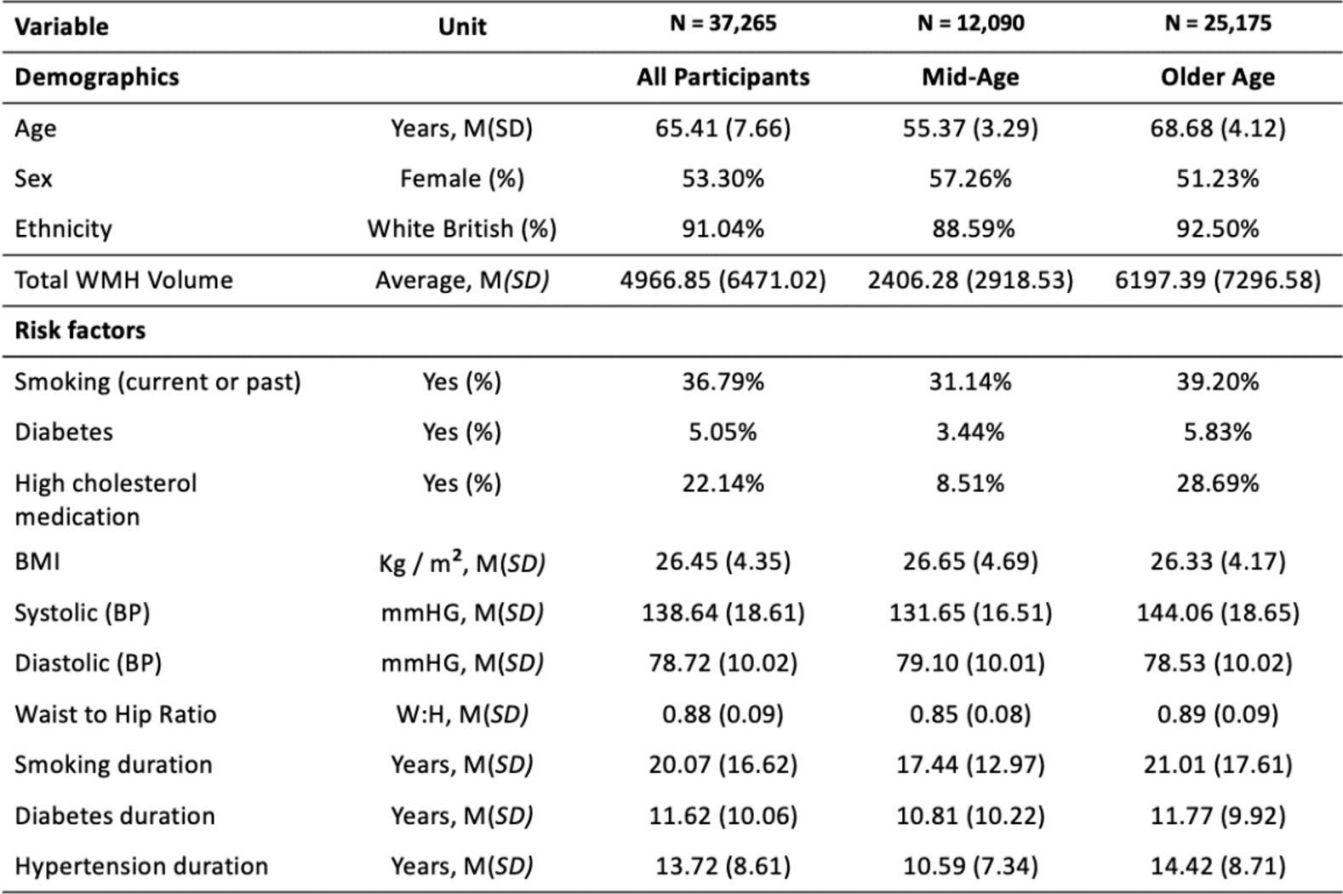
Demographic & cerebrovascular risk factor descriptive statistics. Continuous variables are represented as mean (standard deviation) and binary variables are given as a percentage for all participants and stratified into mid-age and old-age groups.

**Table 2:**
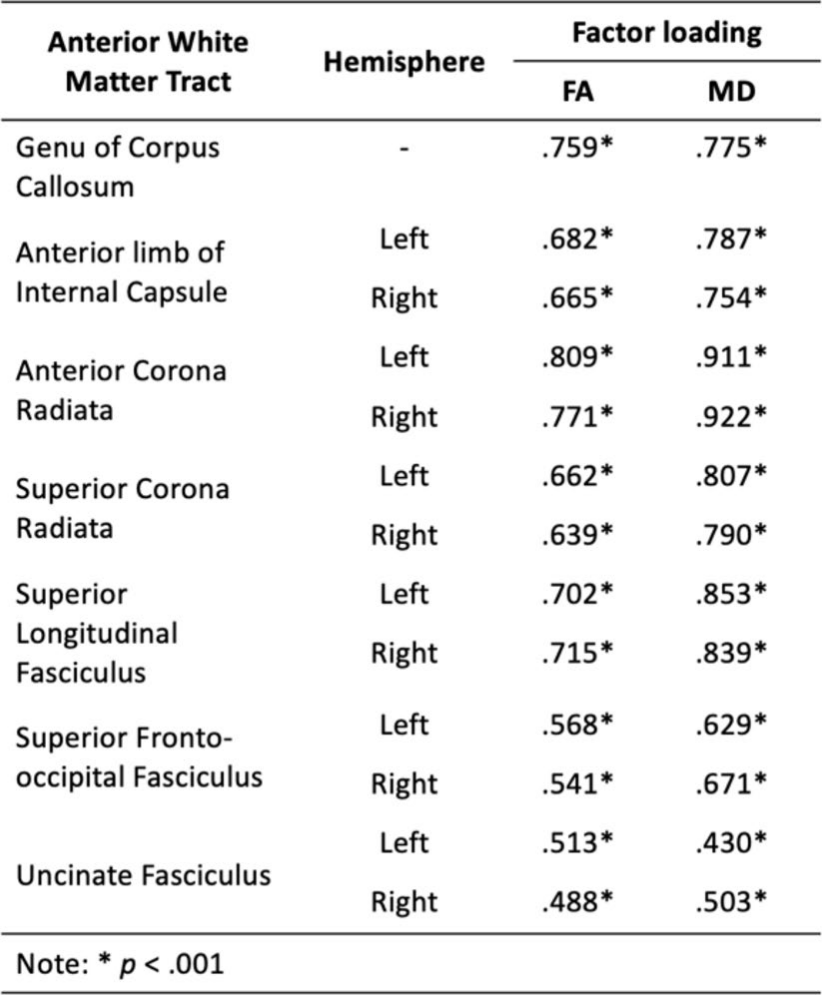
Factor loadings for anterior white matter integrity latent constructs, respectively. Table contains loadings for anterior tracts for both fractional anisotropy (FA) and mean diffusivity (MD).

**Table 3:**
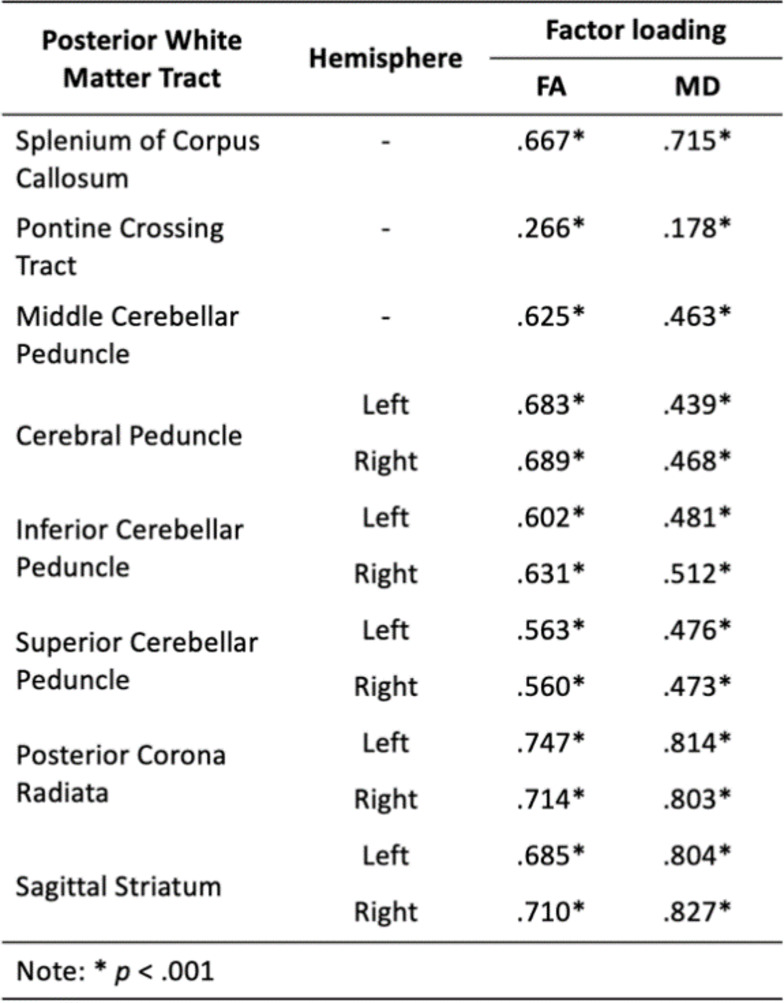
Factor loadings for posterior white matter integrity latent constructs, respectively. Table contains loadings for anterior tracts for both fractional anisotropy (FA) and mean diffusivity (MD).

### **Principal** component analyses **(PCA)**

**Fig. 2:**
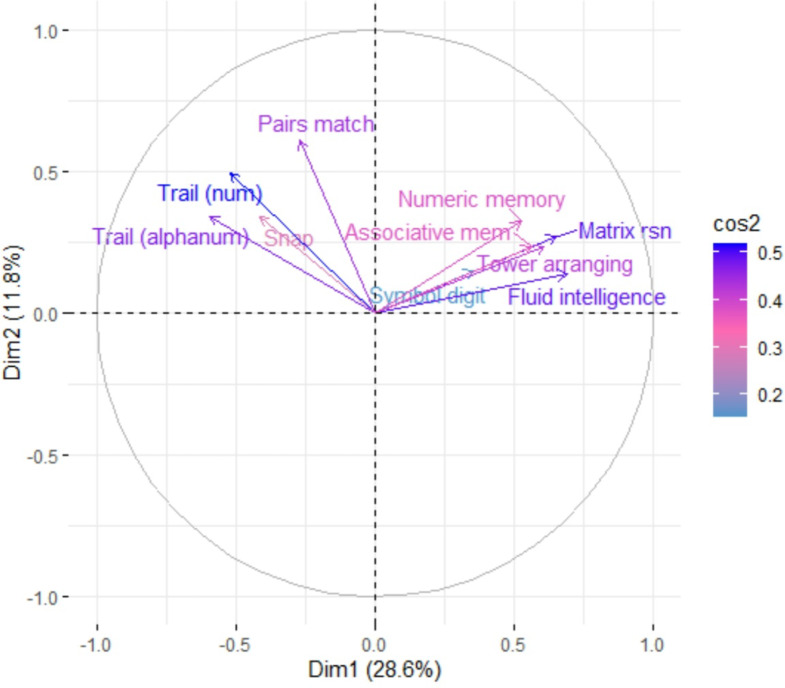
Correlational plot illustrating the relationship between individual cognitive tasks and the first 2 principal components and the quality of variable representation. Principal component 1 is represented on the X-axis and principal component 2 is represented by the y-axis. The gradient colour bar reflects the squared cosine coordinates, which indicates the quality of representation of each cognitive task on their respective principal component. Figure was created using ‘FactoExtra’ package in R.

A PCA derived two principal components, which were subsequently used as latent constructs in the SEMs. The two components and their variable loadings are illustrated by the variable correlation plot in Fig. 2. Here, positively correlated variables are grouped together, with the *X* and *Y* axis representing principal component 1 and 2, respectively. The quality of representation of the variables on the factor map is represented by the colour gradient (squared cosine coordinates – ‘*cos2’*), this illustrates how well a variable reflects a given component. Variables that are coloured in a darker shade of the gradient spectrum reflect a high value, indicating that the variable is a good representation of the component.

**Fig. 3:**
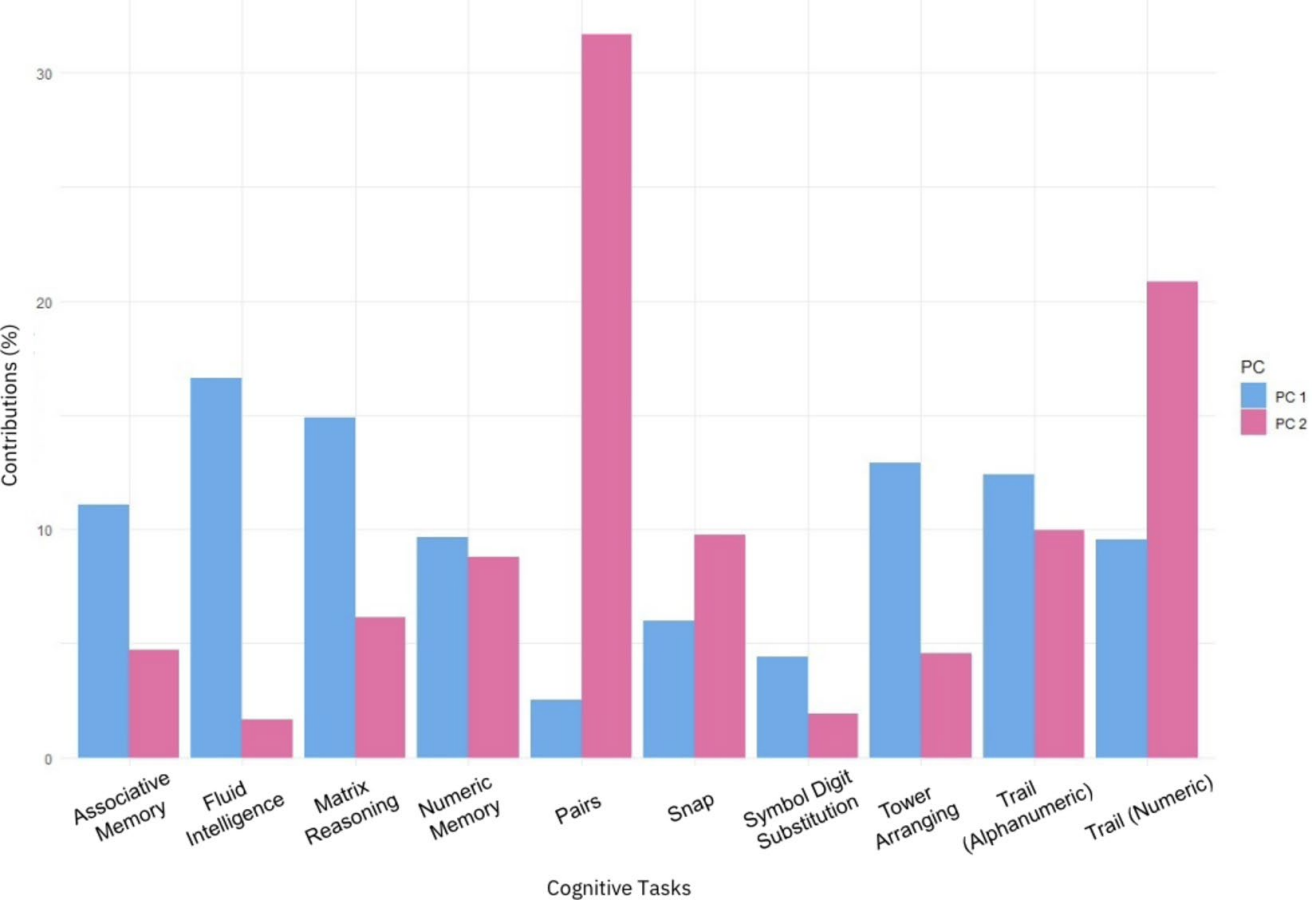
Contributions (%) of each individual cognitive task to principal component 1 & 2. Principal component 1 & are depicted in blue and pink, respectively.

The contribution of each task to principal components 1 & 2 is shown in Fig. 3. Based on this analysis and the pattern of task-types associated with each principal component, we suggest principal component 1 reflects general cognitive function, while principal component 2 reflects processing speed. We drew this conclusion by examining the tasks and measures most highly associated with each principal component. For example, the tasks most highly associated with principal component 1 are Fluid Intelligence, Matrix Reasoning and Tower Arranging. These tasks reflect general cognitive function, as they draw on a number of different cognitive processes, such as abstract reasoning, attention and spatial perception and these are reflected by performance metrics. In comparison, the tasks that loaded most highly on principal component 2 are Pairs, Trail Making (numeric) and Snap. For each of these tasks, processing speed is the dominant cognitive process, and speed metrics were derived from them as measures of performance.

### Structural equation modelling **(SEM)**

**Fig. 4:**
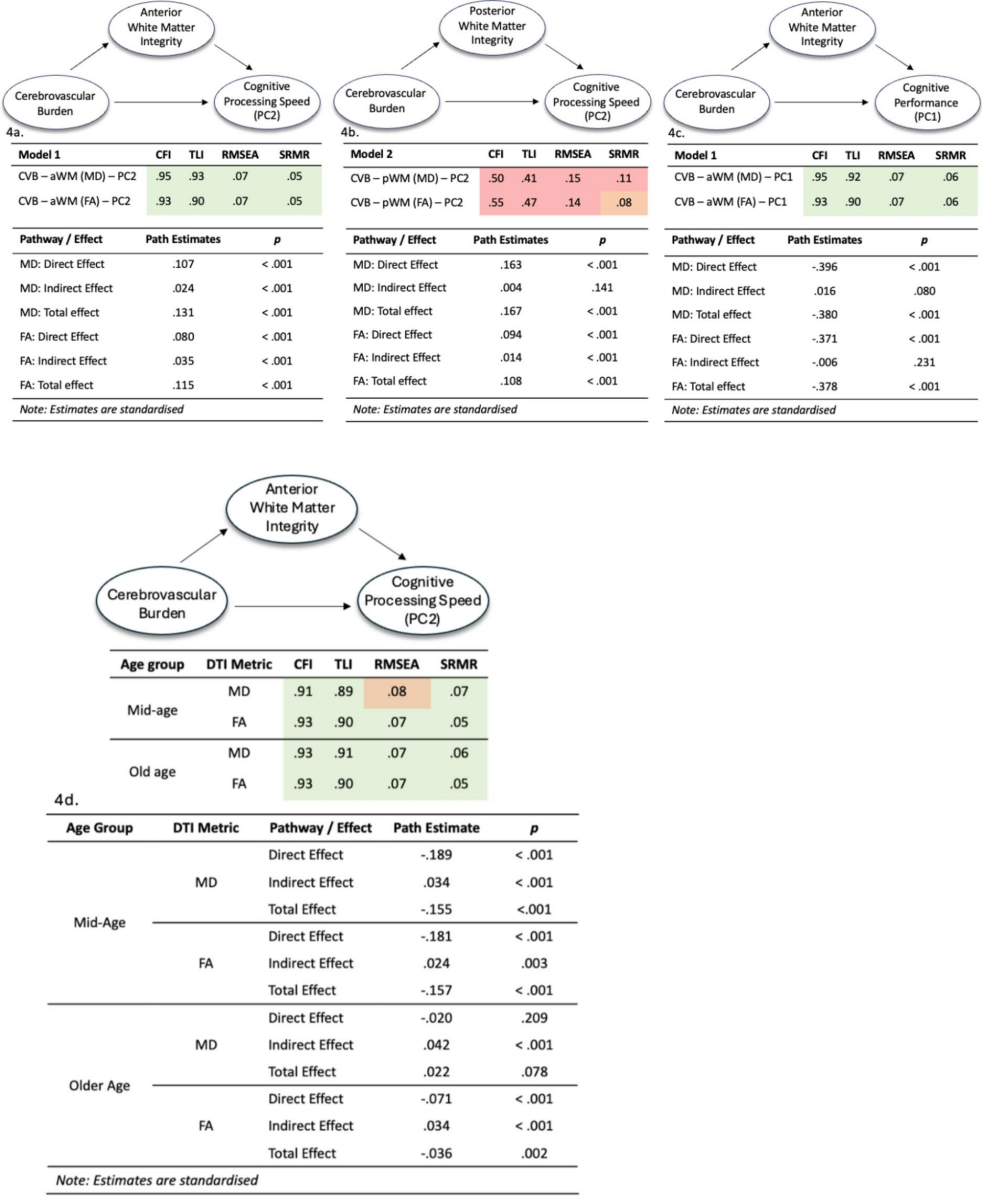
(**a**–**d**) Structural equation models, path loadings and model fit indices. Each figure exhibits a SEM path diagram, standardised path estimates and model fit indices for each of the three models – baseline model, posterior WMI model, general cognitive performance model and baseline model separated into mid- and older age, respectively. Models were deemed to be a good fit based on the following cut off points: 0.90 or above (CFI & TLI) or 0.08 or below ( RMSEA & SRMR). Values highlighted in green indicate good fit, red indicate bad fit and orange indicates borderline. CFI – comparative fit index, TLI – tucker lewis index, RSMEA – root mean square error of approximation, SRMR – standardised root mean square residual.

Once both the latent construct for cerebrovascular burden and anterior WMI were created, they were loaded into a mediation model alongside the cognitive processing speed principal component. Here, a significant direct effect was found, with increased cerebrovascular burden associated with slowed cognitive processing speed (FA & MD: *p* < .001). The indirect effect of this model was also significant, demonstrating that anterior WMI loss partially mediated the relationship between cerebrovascular burden and processing speed (FA & MD: *p* < .001). Model fit indices indicating goodness of fit are shown in Fig. 4a, alongside path and significance values. The proportion of the model explained by the mediator variable for FA & MD was 31% & 18%, respectively. Models were repeated excluding participants with a large burden of white matter pathology and results were unchanged (data not shown).

### Establishing the regional specificity

Regional specificity of the relationship between WMI and the processing speed latent construct was tested by replacing anterior WMI with posterior WMI. In this new model the mediation effect was either no longer significant (MD) or remained significant but reduced (FA), relative to the baseline model (Fig. 4b). The model containing posterior WMI also showed a significantly poorer fit, when compared with the baseline model (*p* < .001; see Fig. 4b). The proportion of the model explained by the mediator variable for FA & MD was 12% & 2%, respectively.

### Establishing the cognitive specificity

To test the cognitive specificity of this effect to processing speed, the outcome measure of the processing speed principal component was replaced with the general cognitive performance principal component. The direct effect between cerebrovascular burden and cognitive outcome remained significant (FA & MD: *p* < .001), showing that greater cerebrovascular burden was associated with worse general cognitive performance. However, the indirect effect no longer significant (MD & FA) as shown in Fig. 4c. This demonstrates no mediatory effect of anterior WMI between cerebrovascular burden and general cognitive performance. Model fit indices demonstrated good fit, statistically comparable to the fit of the baseline model – which was expected, given that only one variable had changed from the baseline model. The proportion of the model explained by the mediator variable for FA & MD was notably low − 1.5% & 4%, respectively.

### Examining effects between mid-age and older-age groups

To examine age-related effects within the baseline SEM models, the data was divided into mid-age and older age groups and the model was reapplied to both across both DTI metrics (FA & MD). A significant, direct effect between cerebrovascular burden and processing speed was found across all metrics and age groups (*p* < .001), with the exception of the older age, MD model *(p = .209).* The total model effects showed a similar pattern, whereby all models were significant, but the older age, MD model was not (*p =* .078). Indirect effects, showing the mediatory effect of anterior white matter integrity were significant across all groups and metrics. The proportion of the model explained by the mediator for the mid-age group for FA & MD is 15% and 22%, respectively. In the older group, the mediator in the FA model explained 94% of the variance within the model. Given that the older-age, MD model shows only a significant indirect effect, this provides evidence for a full mediation in this context. Our results demonstrated good model fit indices across DTI metrics and across age groups, as shown in Fig. 4d, alongside path estimates and significance values.

### Multiple regression analyses

Two multiple regression analyses were conducted to examine the contribution of individual risk factors in predicting processing speed performance and to assess whether additional variance in this relationship can be explained by accounting for risk factor duration. In model 1, *systolic & diastolic blood pressure*,* BMI*,* waist to hip ratio*,* smoking status*,* diabetes status* and *cholesterol* were used to predict *processing speed performance.* Model 2 was identical to model 1, but ‘*smoking status*’ was replaced by *‘smoking duration’* which reflects the number of years participants have smoked (pack years) and ‘*diabetes status*’ was replaced with *‘diabetes duration’* which reflects the number of years since participants received a diabetes diagnosis, where relevant. Model 2 also has an added variable of ‘*hypertension duration*,*’* indicating the number of years since participants received a hypertension diagnosis. Processing speed performance remained the outcome variable in model 2. Sex, Townsend Deprivation Index, and ethnicity were also included in both models as covariates. A smaller subset of the sample (N = 22,475) was used in the regression analysis, as risk factor duration data was not available for a proportion of the sample who had indicated they had a given risk factor.

### Overall model effects

Model 1, containing static risk factor information, demonstrated significant effects (*F(*29, 22475) = 33.62, *p* < .001, *R²* 0.042), whereby high levels of burden were associated with slower processing speed. Model 2, *with* the addition of duration variables for *hypertension*,* diabetes* and *smoking* alongside static measures for the other risk factor variables, also demonstrated significant effects (*F(*33, 22474) = 34.51, *p* < .001, *R²* 0.046). Similarly, higher levels of burden and longer risk factor duration was associated with slower processing speed. The difference between the two models was significant, with model 2 explaining a greater amount of variance (*R²* 0.002), relative to model 1 (ANOVA; F(1, 22475) = 99.13, *p *< .001). This suggests risk factor duration explains a unique proportion of the variance and improves the ability of cerebrovascular burden to predict processing speed performance.

### Individual risk factor effects

**Fig. 5:**
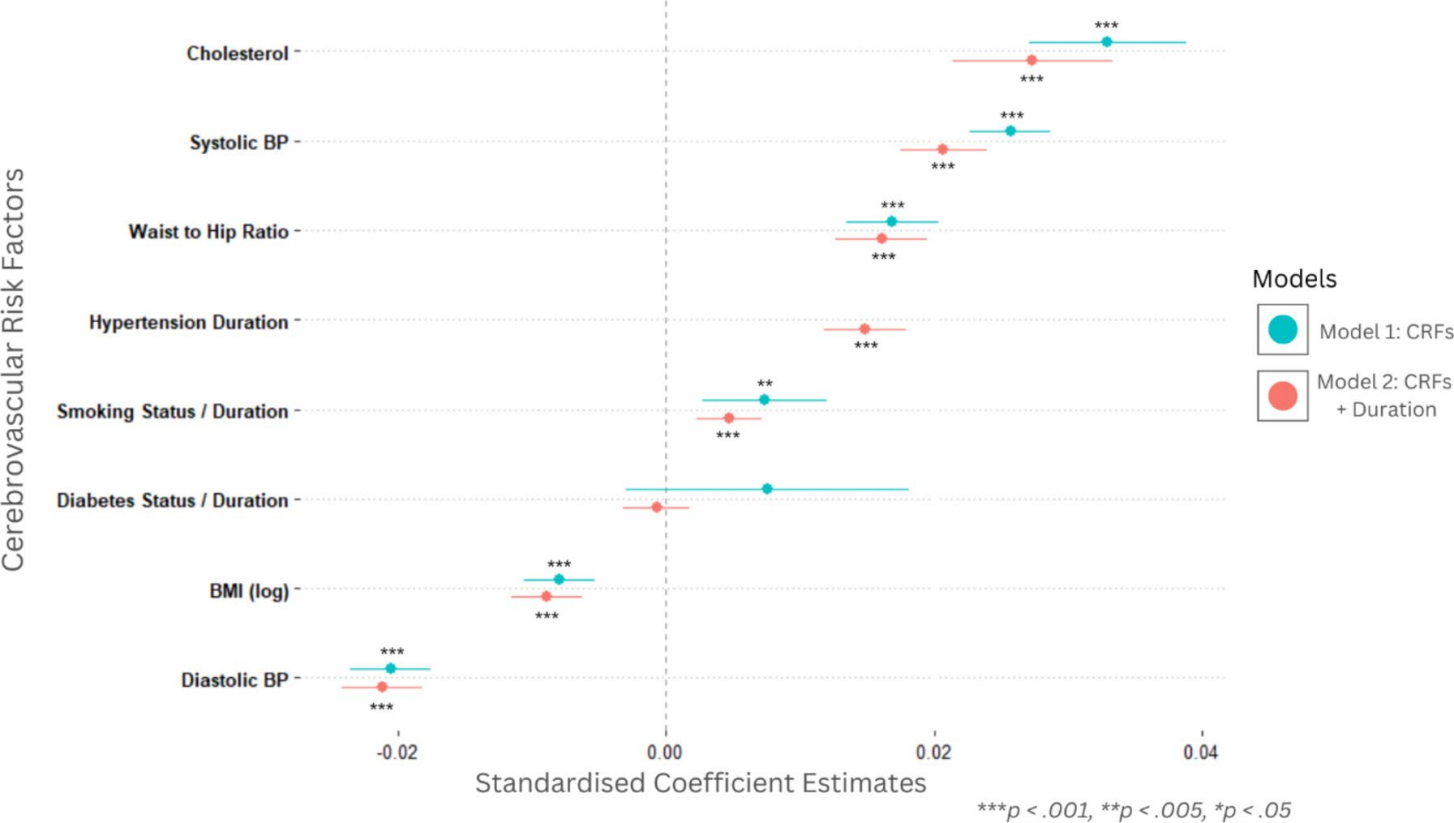
Cerebrovascular risk factors (model 1) and risk factor duration (model 2) predicting processing speed performance. Model 2 depicts the relationship between individual cerebrovascular risk factors and processing speed. The second replicates model 1, with the addition of risk factor duration for Hypertension, Diabetes and Smoking. Note: ‘Diabetes’ in model 1 is a binary yes/no variable based on diagnosis, but in model 2, this reflects how long (in years) participants have had a diagnosis in years. Similarly, ‘Smoking’ in model 1 is binary - indicates whether participants are current or past smokers / never smoked and model 2 reflects number of pack years.

All variables, apart from *diabetes* in both model 1 and model 2 were significant predictors of processing speed performance (as derived from *Principal Component 2*), each explaining unique variance. The largest amount of variance was explained by *Cholesterol* in both model 1 (*β*= 0.033, σ = 0.003) and model 2 (*β* = 0.027, σ = 0.003). This was followed closely by both *systolic* (model 1 - *β*= 0.026, σ = 0.002; model 2 - *β* = 0.021, σ = 0.002) and *diastolic* (model 1 - *β*= − 0.021, σ = 0.002; model 2 - *β* = − 0.021, σ = 0.002) blood pressure. The lowest significant contributors in the model were *Smoking* (model 1 - *β*= 0.007, σ = 0.002; model 2 - *β* = − 0.003, σ = 0.001) and *BMI* (model 1 - *β*= − 0.008, σ = 0.001; model 2 - *β* = − 0.009, σ = 0.001). The regression coefficients for each risk factor in each model are shown in Fig. 5. *All reported coefficients are standardised.*

In model 2 hypertensive duration was found to be a significant predictor (model 2 - *β* = 0.015, σ = 0.002), and comparable to established risk factors, such as BMI. Number of pack years was also a significant predictor (model 2 - *β* = 0.005, σ = 0.001), performing comparably to static measures of smoking status, shown in model 1. In contrast replacing static measures of diabetes with duration measures had less (non-significant) effect. The findings suggest that accounting for risk factor duration, primarily hypertension duration, significantly improves the prediction of processing speed from cerebrovascular burden, relative to static burden models that do not account for risk factor duration.

## Discussion

In a large sample of people without current or previous neurological disease, we found that cerebrovascular burden was associated with poorer cognitive function and regionally specific white matter pathology. More specifically, we found that increased cerebrovascular burden was associated with slowed processing speed – and that this effect was mediated by anterior WMI. Our findings demonstrate this effect in the overall sample, but also in both mid-age and older age groups, which highlighted that mediatory effects of anterior WMI are stronger in older age. The mediatory role of WMI was either diminished (FA) or no longer significant (MD) when anterior tracts were replaced with posterior tracts. Additionally, when processing speed was replaced with a metric of general cognitive performance the mediatory effect of anterior WMI was no longer significant (consistent in both FA & MD models). Together, these findings demonstrate the regionally specific mediatory effect of anterior WMI on the relationship between cerebrovascular burden and processing speed. Furthermore, we demonstrate the relative impact of cerebrovascular risk factors and risk factor duration on cognitive processing speed performance. Each individual cerebrovascular risk factor, including systolic & diastolic blood pressure, smoking status, cholesterol, BMI and waist-to-hip ratio significantly predicted processing speed performance and each contributed unique variance. This is with the exception of diabetes which showed no significant predictive effects. This may be reflected by the absence of data to separate type 1 and type 2 diabetes in the UK Biobank. In addition, accounting for risk factor duration for hypertension, smoking and diabetes, was shown to improve predictions of processing speed performance, beyond the inclusion of static cerebrovascular risk factor measures alone. These findings support our initial predictions, that a pathway, through which cerebrovascular risk factors impact cognitive processing speed, is selectively mediated by alterations in anterior WMI and that cerebrovascular burden can better predict processing speed when duration is accounted for, alongside static measures.

Our findings add to the spectrum of vascular diseases and risk factors linked to heightened susceptibility of anterior white matter pathology, such as atrial fibrillation^28^, subcortical ischaemic vascular disease^29^, vascular dementia^30^ and cerebrovascular risk factors^15,31^, highlighting the specific role of cholesterol, blood pressure, smoking, diabetes, BMI and waist to hip ratio. The pattern of anterior-focused white matter change shown, is also notably similar to the regional ischemic vulnerability of the brain to hypoperfusion shown by clinical stroke samples^32^. While the mechanisms underpinning this regionally specific vulnerability are incompletely understood, it likely has a vascular basis^33^. One proposed mechanism is that anterior regions are particularly susceptible to haemodynamic derangement, whereby the vasculature undergoes structural changes and arterioles become tortuous or coiled^30,34^ – and further exacerbated by cerebrovascular burden^35^. Consequently, this likely reduces local blood flow and limits white matter perfusion, leading to the emergence of white matter pathology^36^. However, further work is required to unpick the cellular, haemodynamic, and vascular mechanisms underpinning the relationship between cerebrovascular burden and anterior white matter alterations. Our findings also demonstrate some variability between DTI metrics, FA and MD – whereby the indirect effect of posterior WMI remained significant but modest for FA, but MD was non-significant. The finding of changes in FA without concomitant significant change in MD is difficult to explain with certainty but may reflect variances in tissue and pathological sensitivity.

We also demonstrate the cognitive consequences - particularly for processing speed - of cerebrovascular burden via breakdown of anterior WMI. This link between anterior WMI and slowed processing speed is consistent with findings from a range of white matter-affected diseases, such as multiple sclerosis^37^, Alexander disease^38^ and cerebral small vessel disease^20^. There are several mechanisms that likely drive this affect, such as myelin loss, axonal integrity alterations and other white-matter pathological processes indicated by various diffusion metrics. These factors likely prevent, or slow, neural signalling, ultimately resulting in slowed cognitive processing speed^39^. This view is further supported by previous associations between the presence of highly myelinated tracts and faster processing speed^40^. We also demonstrated a link between cerebrovascular burden and more general cognitive performance, which was not well explained by anterior WMI – whereby the model was significant, but this was primarily driven by the direct effect, as the mediatory effect of anterior WMI was not significant for FA or MD. Different white matter networks may be impacted by cerebrovascular burden and may better explain changes in other cognitive domains and highlights the preferential contribution of anterior WMI to processing speed performance.

Our work also highlights age-related variation in the mediatory effect of anterior white matter integrity between cerebrovascular health and processing speed. As highlighted, indirect effects are significant across all groups and DTI metrics, but the mediator explains much greater variance in the older group, with the FA model showing a full mediation effect. The ageing process itself can act as vascular risk factor, by driving functional and structural changes in the cerebrovascular network – such as tortuous arterioles, capillary loss and reduced blood flow^36^. As a result, the vascular system becomes less efficient with increasing age and therefore, the effects of cerebrovascular burden are amplified – this may explain the stronger mediatory effect in the older age group.

Our findings illustrate the relative and unique contribution of individual risk factors in predictive processing speed performance. Most notably, the highest contributors were cholesterol and systolic blood pressure. While the specific mechanisms driving this link are unclear, cardiometabolic risk factors, such as systolic blood pressure and cholesterol have a direct influence on cerebral blood flow 13, which is vital for the function and health of neural networks. Poor cerebrovascular blood flow also compromises white matter networks 13, which we have shown mediates the relationship between vascular health and processing speed. Our findings also offer practical suggestions to improve cerebrovascular burden quantification. Traditional methods of cerebrovascular risk quantification used in clinic and research assess relative cerebrovascular burden from a static, cross-sectional viewpoint and commonly use methods such as the Framingham Risk score^41^ and QRISK^42^. Here we show, for the first time in one model, that modelling risk factor duration, specifically for hypertension and diabetes duration and smoking pack years, can significantly improve the extent to which cerebrovascular burden predicts processing speed – though, it must be noted that these effects are modest. Such findings suggest traditional methods may underestimate the extent of cerebrovascular burden in health and disease. Although risk factor duration metrics were participant-estimated, our findings align well with previous work. For example, smoking duration and intensity has been found to negatively predict cognitive function^43^. In addition, previous work used clinic-confirmed hypertensive duration metrics to highlight a negative relationship with cognitive function^25^. These findings converge to suggest that participant estimates of risk factor duration usefully enhance predictions of cerebrovascular burden on cognitive function. Therefore, including this information, where available, will lead to more sensitive predictive models of brain health and cognitive function.

## Limitations and future research directions

Despite the large-scale sample and quality of the UK Biobank data, there are several methodological limitations to consider. First, we used cross-sectional data, which is limited by several caveats – including the inability to confidently claim causal inference. However, the inclusion of risk factor duration metrics in our work provides an estimate of longitudinal insight into the body-brain-cognition relationships described, indicating burden builds over time for individual risk factors. Future work should aim to extend our findings, by assessing whether risk factor duration also influences white matter pathology across age groups, as well as processing speed. The schedule of re-imaging currently underway by the UK Biobank will allow longitudinal analyses and complement our findings on cerebrovascular change and cognition. Second, in order to optimise the data available, we employed a PCA imputation algorithm to effectively handle missing data across several cognitive tests. While the imputed data only accounts for a very small percentage of the total data (< 5%), this data is estimated and so, this must be highlighted as a limitation within our study. Third, those who opted to participate in the UK Biobank tend to represent less socioeconomically deprived areas, with fewer morbidities than the rest of the UK, with comparatively lower rates of obesity and smoking^44^. Although this may at least slightly limit the generalisability of the results, our findings were derived from a relatively healthy population, suggesting that these effects may in fact be exacerbated in a more representative sample. In addition to this, while the UK Biobank offers excellent phenotyping, some elements of past medical history are incomplete. For example, it is not clear how long participants may have received medication for diabetes or hypertension in the past, unless they are currently taking those medications. Future work should assess the influence this has on cognitive and brain structure outcomes. Fourth, we specifically studied white matter changes in anterior and posterior regions using FA & MD MRI metrics, but it is likely that other, more granular white matter changes (including regions such as the cingulum) may also be relevant for understanding various aspects of cognition. Additionally, different diffusion measures of white matter which were not included in the current study may also offer further insight, beyond our findings. For example, measures such as NODDI, radial or axial diffusivity. Lastly, though we did control for sex, TDI and ethnicity, we did not control for education, which may also contribute to the relationships found.

In summary, our work proposes a pathway through which cerebrovascular burden impacts brain integrity and cognitive function, providing evidence of both regional and cognitive specificity. We show that greater cerebrovascular burden was associated with slowed processing speed – an effect at least partially mediated by alterations in anterior WMI, which strengthens with age. We demonstrate that we can used cerebrovascular risk factors to predict processing speed performance – with cholesterol and blood pressure being the most prominent predictors. However, we also showed that models which include risk factor duration alongside static risk factors, can improve predictive outcomes. These findings are shown in a large, generally healthy cohort, where vascular insult is relatively mild. Addressing these risk factors as early as possible will likely aid in the prevention of later-life clinically significant cognitive decline. Through future work we can establish the pace at which these structural and cognitive changes occur – and the time windows during which effective interventions can optimally be applied.

## Methods

### Participants

Participant’s data were obtained from the UK Biobank’s latest release, as of March 2021 (application number: 76847). Participants with missing cognitive, cerebrovascular and neuroimaging data were excluded. In addition, participants were excluded if they presented with a history or current diagnosis of neurological disease, head/brain injury or trauma, stroke, TIA, alcohol or opioid dependence, Epilepsy, Parkinson’s disease, Alzheimer’s disease, Dementia, cognitive impairment, chronic or degenerative neurological issues (including demyelination-associated syndromes), brain hematoma, brain abscess, meningitis, encephalitis, infection of the nervous system, haemorrhage or multiple sclerosis during their medical interview. As outlined in the non-cancer illness codes (https://biobank.ndph.ox.ac.uk/showcase/coding.cgi?id=6). In total, data from 37,265 participants were used in the main analysis, aged 40–75. A smaller subset of the sample (N = 22475) was used in the regression analysis, due to missing risk factor duration data. Ethical approval was granted to the UK Biobank by the North-West Multi-centre Research Ethics Committee and adheres to the regulations and guidelines outlined in the *‘UK Biobank Ethics and Governance Framework’ - REC code 21/NW/0157*. All participants provided informed consent for their anonymised data to be used.

### Cognitive testing

Computerised cognitive testing was administered using a touch-screen during the MRI visit, completed outside of the scanner. The cognitive tests analysed were tests of numeric memory, fluid intelligence, trail making (numeric and alphanumeric), matrix pattern completion, symbol digit substitution, tower rearranging, paired associative learning, pairs matching and a reaction-time based game of snap. Detailed summaries of each individual cognitive assessment undertaken during the neuroimaging visit can be found elsewhere and in UK Biobank documentation (https://biobank.ctsu.ox.ac.uk/crystal/label.cgi?id=100026).

### MRI data acquisition and analysis

Imaging data for the Biobank were collected using a Siemens Skyra 3T Scanner and 32-channel head coil, per the openly available protocol and acquisition detail documentation (https://biobank.ctsu.ox.ac.uk/crystal/crystal/docs/brain_mri.pdf & https://www.fmrib.ox.ac.uk/ukbiobank/protocol). In short, the dMRI acquisition consisted of a 2 mm isotropic spin-echo multiband echo-planar sequence, with 50 *b* = 1000s mm and 50 *b* = 2000s mm diffusion weighted volumes (100 encoding directions total).

**Fig. 6:**
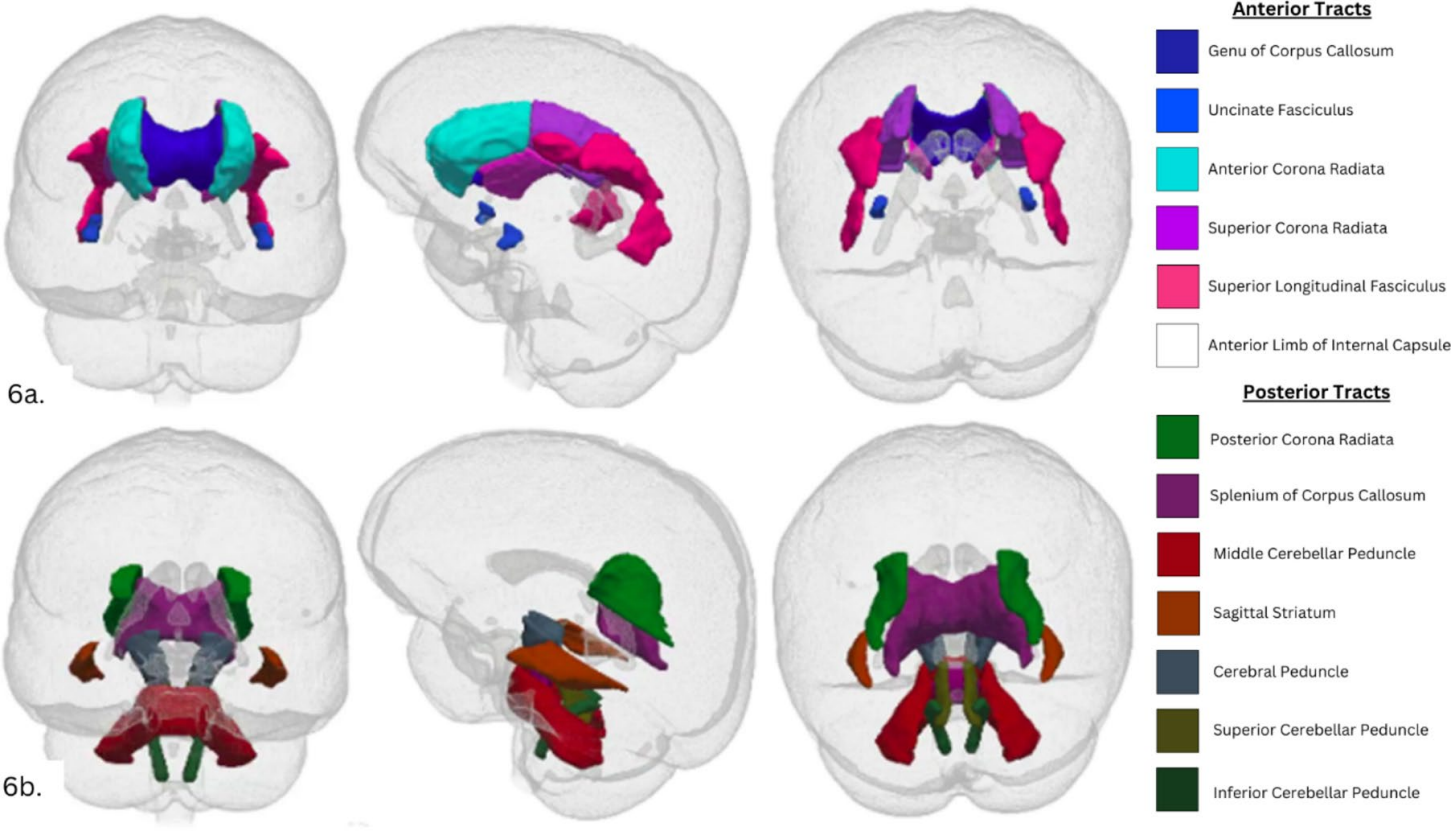
Anterior (**a**) and posterior (**b**) white matter tracts reflect the tracts loaded onto each latent construct of white matter integrity, respectively.

All imaging data were quality checked internally by the UK Biobank team, before being processed by an openly available automated pipeline^45^. One component of this pipeline is the generation of image-derived phenotypes (IDPs), which are individual tract or volumetric averages of specific regions in the brain. The IDPs of interest, used in the current analysis were tract-averaged fractional anisotropy (FA) and mean diffusivity (MD) metrics for the following anterior white matter tracts: genu of corpus callosum, anterior corona radiata, superior corona radiata, anterior limb of internal capsule, superior fronto-occipital fasciculus, superior longitudinal fasciculus, uncinate fasciculus (as shown in Fig. 6a*)*. The control, posterior tracts used were cerebral peduncle, inferior cerebellar peduncle, middle cerebellar peduncle, superior cerebellar peduncle, posterior corona radiata, splenium of corpus callosum, sagittal stratum and pontine crossing tract (shown in Fig. 6b*)*.

### Cerebrovascular burden

**Fig. 7:**
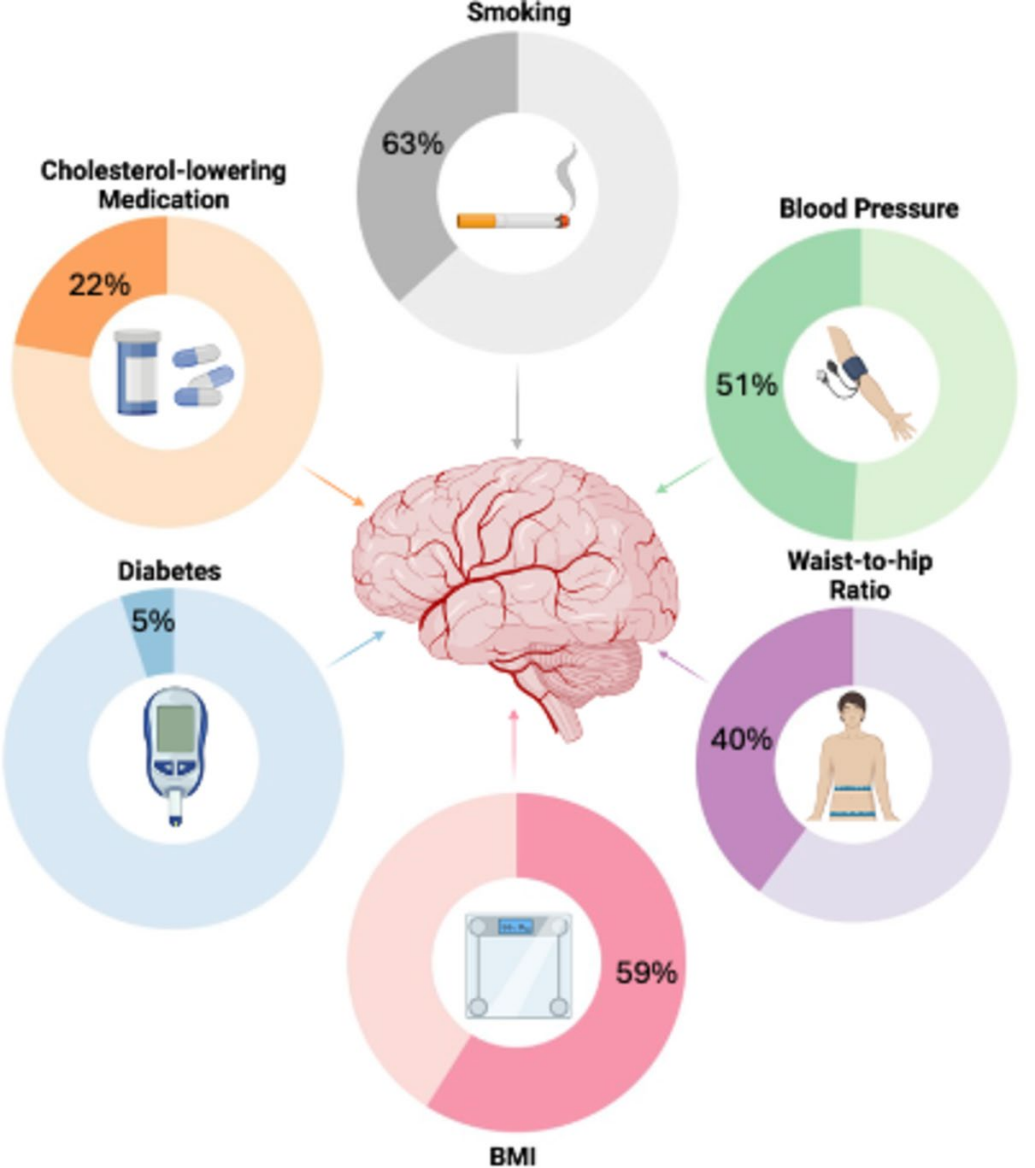
Prevalence of individual cerebrovascular risk factors in the sample. This figure depicts the proportion of the sample with each individual risk factor – indicated by the darker proportion of each circle. To be considered to have a given risk factor, they needed to meet the following criteria for each risk factor – smoking: indication of current or past active smoking status, blood pressure: 140/90mmHg, waist-to-hip ratio: females - < 0.80, males - <0.90, BMI: >25, Diabetes: diagnosis confirmed by a doctor, cholesterol: currently taking cholesterol lowering medication. Figure was created used BioRender.com.

Cerebrovascular risk factor information was collected from participants during a medical history interview, during their neuroimaging session. Where continuous variables were unavailable, binary risk factors were coded based on the presence of a given risk factor, described in detail below. Figure 7 depicts the proportion of the sample with any given risk factor.

*Smoking*—Participants who indicated current or past active smoking status were coded with a score of 1, those who did not were coded with a score of 0.

*Diabetes*—Participants who indicated that they had received a diabetes diagnosis were coded with a value of 1 and those who had not, were coded with a value of 0.

*Hypercholesterolaemia*—A current prescription of cholesterol-lowering medication was used as a surrogate measure for high cholesterol and participants who indicated that they take cholesterol-lowering medication were coded with a value of 1 and those who did not, a value of 0.

*Blood pressure*—Blood pressure (BP) was measured twice, moments apart using an automated Omron digital BP monitor for both diastolic and systolic BP, which were then averaged for each.

*Waist-to-hip ratio & BMI*—Anthropometric measures were taken manually once participants had removed any bulky clothing. Waist and hip circumference measures were transformed into a ratio metric by dividing the waist hip measurements. BMI was calculated by dividing weight (kg) by the square of height in metres.

### Risk factor duration

Risk factor duration was calculated for each risk factor, where available, specifically - hypertension, smoking and diabetes.

*Hypertensive duration*—Participants who had indicated that they were currently on hypertensive medication were asked to estimate the age they were diagnosed with high blood pressure, which was subtracted from their current age.

*Smoking duration*—To quantify how long each participant with a positive smoking status had been smoking, pack years was calculated by dividing the number of cigarettes smoked per day by 20, multiplied by the number of years smoking.

*Diabetes duration*—Participants who indicated that they were diabetic were asked to estimate the age they were diagnosed with diabetes, which was subtracted from their current age.

### Statistical analyses

Firstly, data were visually inspected to assess the distribution of each variable and to ensure they met the necessary parametric assumptions. Participants with IDPs = 0 were omitted entirely from the dataset, based on a priori reasoning that their data may be missing due to scan artefacts or other technical issues. BMI data were log-transformed, to correct for skewed distribution. Any participants with missing cerebrovascular risk, cognitive or neuroimaging were omitted from the dataset & analysis.

A principal component analysis (PCA) was performed to derive two new latent constructs from all cognitive data available from the UK Biobank during the participants’ imaging visit. This approach is particularly useful in this instance, as it allows us to compress data from a large breadth of correlated cognitive tasks into two principal components. The orthogonal projection of data optimises the unique variance explained by the new principal components. With the exception of data from pilot tests, tests with significant amounts of missing data and those unavailable for download were not included. Due to the volume of missing cognitive data from the UK Biobank, the ‘*MissMDA’* package was used to obtain scores and PCA loadings^46^. This package allows PCA to be performed on datasets with missing values, by using an iterative PCA algorithm to impute missing data and perform the PCA. We then used the factor loadings from PC1 and PC2 in our SEMs. Based on the factor loadings, we described PC1 to reflect more general cognitive performance, as tasks such as associative memory, tower rearranging, matrix pattern completion and fluid intelligence loaded highly, which all measured performance. We described PC2 to represent cognitive processing speed, as the cognitive tests trail making, pairs matching and snap loaded highly, which were measures of speed.

In preparation for the SEM, a latent construct of cerebrovascular burden was created, loaded with each cerebrovascular risk factor using confirmatory factor analysis. This method of burden quantification has been validated in previous studies and provides a more informative indicator of body-brain relationships, relative to commonly used aggregate quantification methods^14,15^. Similarly, two latent constructs of anterior WMI were also created using confirmatory factor analysis, with left & right tracts loaded separately for each construct - one representing FA and the other, MD. In both cases, using confirmatory factor analysis allows us to reduce noise, by capturing shared variance across variables within each latent construct, unlike other methods, such as averaging, which would potentially amplify noise contributions. Using this approach, we are also able to show fit indices - demonstrating how well the variables fit within the latent construct.

A SEM approach was used to model and quantify the multivariate, directional relationship between cerebrovascular burden, white matter integrity and cognition, based on a-priori hypotheses. This approach also allowed us to derive latent construct and model fit indices. In the SEM, the latent construct of cerebrovascular burden (loaded with cerebrovascular risk factors, as described previously) acted as the predictor variable. The latent construct of anterior WMI acted as the mediator variable. Two separate models were created - one containing FA and the other, MD. The principal component for processing speed was used as the outcome variable for the baseline SEM. The ‘*lavaan 0.6–12’* package^47^ was used to create the latent constructs using confirmatory factor analysis and to build and analyse the SEMs. We also further wanted to assess how the baseline model performs across different age groups. The data were divided into mid-age (60 or below, N = 12,090) and older age groups (above 60, N = 25,175). These models were identical to the baseline model and were used to assess the mediatory effect of anterior WMI between cerebrovascular burden and cognitive processing speed (PC2), which was performed separately for the two age groups.

To establish the regional specificity of this effect, a latent construct of posterior WMI was also created. A new SEM was built, identical to the baseline model, but the anterior WMI mediatory variable was replaced with the posterior WMI latent variable. To explore cognitive specificity, a third SEM was built, but here the outcome variable was replaced with the principal component for general cognitive performance, rather than processing speed. Ethnicity, Townsend Deprivation Index (TDI) & sex were added as covariates onto each path in all SEM models as control measures. Townsend Deprivation Index is a validated measure of material deprivation, accounting for car ownership, employment, home ownership and household over-crowding, provided by census data for a given geographical area. The proportion of variance explained was calculated for each SEM model by dividing the indirect effect by the total effect and multiplying the output by 100.

Two multiple regression analyses were also performed to characterise the relative relationship between individual risk factors, and processing speed and to assess whether this relationship can be strengthened by accounting for risk factor duration. In model 1, all cerebrovascular risk factors were used as predictor variables (namely, *systolic & diastolic blood pressure*,* BMI*,* waist to hip ratio*,* smoking status*,* diabetes status* and *cholesterol*) and processing speed (PC2) acted as the outcome variable, To assess whether accounting for risk factor duration can improve the predictive utility of cerebrovascular burden on processing speed (PC2), available risk factor duration data was added to form model 2. Model 2 was identical to model 1, but *smoking duration* and *diabetes duration* replaced *smoking status* and *diabetes status*, to avoid redundancy in the models. *Hypertension duration* was also added to model 2, alongside static risk factors from model 1, namely, *systolic & diastolic blood pressure*,* BMI*,* waist to hip ratio and cholesterol* to predict processing speed performance (PC2). Ethnicity, TDI & sex were added as covariates to both regression models and an age interaction term was added for each of the risk factor duration variables in model 2. The two models were statistically compared.

## Data Availability

All UK Biobank information is available online on the webpage www.ukbiobank. Data access is available through applications. Information about the data available on the UK Biobank database is available via the showcase website - https://biobank.ndph.ox.ac.uk/showcase/.

## References

[1] de la Torre, J. C. Cardiovascular risk factors promote brain hypoperfusion leading to cognitive decline and dementia. *Cardiovasc. Psychiatry Neurol.* **2012**, 367516 (2012). 10.1155/2012/367516 PMC3518077 23243502

[2] Hachinski, V. et al. Preventing dementia by preventing stroke: the Berlin manifesto. *Alzheimers Dement.* **15**, 961–984 (2019). 31327392 10.1016/j.jalz.2019.06.001 PMC7001744

[3] Iadecola, C. The neurovascular unit coming of age: a journey through neurovascular coupling in health and disease. *Neuron* **96**, 17–42 (2017). 28957666 10.1016/j.neuron.2017.07.030 PMC5657612

[4] Dufouil, C. et al. Severe cerebral white matter hyperintensities predict severe cognitive decline in patients with cerebrovascular disease history. *Stroke* **40**, 2219–2221 (2009). 19390070 10.1161/STROKEAHA.108.540633

[5] de Groot, M. et al. Changes in normal-appearing white matter precede development of white matter lesions. *Stroke* **44**, 1037–1042 (2013). 23429507 10.1161/STROKEAHA.112.680223

[6] Pflanz, C. P. et al. Association of blood pressure lowering intensity with white matter network integrity in patients with cerebral small vessel disease. *Neurology* **99**, e1945–e1953 (2022). 35977831 10.1212/WNL.0000000000201018 PMC9620809

[7] Raz, N., Rodrigue, K. M. & Acker, J. D. Hypertension and the brain: vulnerability of the prefrontal regions and executive functions. *Behav. Neurosci.* **117**, 1169–1180 (2003). 14674838 10.1037/0735-7044.117.6.1169

[8] Gao, S. et al. White matter microstructural change contributes to worse cognitive function in patients with type 2 diabetes. *Diabetes* **68**, 2085–2094 (2019). 31439643 10.2337/db19-0233 PMC6804632

[9] Hsu, J. L. et al. Microstructural white matter abnormalities in type 2 diabetes mellitus: A diffusion tensor imaging study. *NeuroImage* **59**, 1098–1105 (2012). 21967726 10.1016/j.neuroimage.2011.09.041

[10] Daoust, J. et al. White matter integrity differences in obesity: A meta-analysis of diffusion tensor imaging studies. *Neurosci. Biobehav Rev.* **129**, 133–141 (2021). 34284063 10.1016/j.neubiorev.2021.07.020

[11] Evans Tavia, E. et al. White matter microstructure improves stroke risk prediction in the general population. *Stroke* **47**, 2756–2762 (2016). 27703085 10.1161/STROKEAHA.116.014651

[12] Kandil, H. et al. Studying the role of cerebrovascular changes in different compartments in human brains in hypertension prediction. *Appl. Sci.* **12**, 4291 (2022).

[13] Beason-Held, L. L., Moghekar, A., Zonderman, A. B., Kraut, M. A. & Resnick, S. M. Longitudinal changes in cerebral blood flow in the older hypertensive brain. *Stroke* **38**, 1766–1773 (2007). 17510458 10.1161/STROKEAHA.106.477109

[14] Veldsman, M. et al. Cerebrovascular risk factors impact frontoparietal network integrity and executive function in healthy ageing. *Nat. Commun.* **11**, 4340 (2020). 32895386 10.1038/s41467-020-18201-5 PMC7477206

[15] Cox, S. R. et al. Associations between vascular risk factors and brain MRI indices in UK biobank. *Eur. Heart J.* **40**, 2290–2300 (2019). 30854560 10.1093/eurheartj/ehz100 PMC6642726

[16] Becker, D., Breustedt, W. & Zuber, C. I. Surpassing simple aggregation: advanced strategies for analyzing contextual-level outcomes in multilevel models. *Methods Data Anal.* **12**, 31 (2018).

[17] Xu, X. et al. Association of magnetic resonance imaging markers of cerebrovascular disease burden and cognition. *Stroke* **46**, 2808–2814 (2015). 26330446 10.1161/STROKEAHA.115.010700

[18] Verdelho, A. et al. Cognitive impairment in patients with cerebrovascular disease: A white paper from the links between stroke ESO dementia committee. *Eur. Stroke J.* **6**, 5–17 (2021). 33817330 10.1177/23969873211000258 PMC7995319

[19] Song, R. et al. Associations between cardiovascular risk, structural brain changes, and cognitive decline. *J. Am. Coll. Cardiol.* **75**, 2525–2534 (2020). 32439001 10.1016/j.jacc.2020.03.053 PMC10061875

[20] Prins, N. D. et al. Cerebral small-vessel disease and decline in information processing speed, executive function and memory. *Brain* **128**, 2034–2041 (2005). 15947059 10.1093/brain/awh553

[21] Kochunov, P. et al. Processing speed is correlated with cerebral health markers in the frontal lobes as quantified by neuroimaging. *NeuroImage* **49**, 1190–1199 (2010). 19796691 10.1016/j.neuroimage.2009.09.052 PMC2789896

[22] Salthouse, T. A. The processing-speed theory of adult age differences in cognition. *Psychol. Rev.* **103**, 403–428 (1996). 8759042 10.1037/0033-295x.103.3.403

[23] Liebel, S. W. et al. Cognitive processing speed mediates the effects of cardiovascular disease on executive functioning. *Neuropsychology* **31**, 44–51 (2017). 27841458 10.1037/neu0000324 PMC5191907

[24] de Leeuw, F. E. et al. Hypertension and cerebral white matter lesions in a prospective cohort study. *Brain* **125**, 765–772 (2002). 11912110 10.1093/brain/awf077

[25] Li, T., Xiang, J., Bai, J., Wang, R. & Zhao, Z. The association of duration of hypertension and changes in cognitive function in hypertension patients. *Zhonghua Nei Ke Za Zhi*. **53**, 278–282 (2014). 24857300

[26] Knopman, D. et al. Cardiovascular risk factors and cognitive decline in middle-aged adults. *Neurology* **56**, 42–48 (2001). 11148234 10.1212/wnl.56.1.42

[27] Gray, J. C. et al. Associations of cigarette smoking with Gray and white matter in the UK biobank. *Neuropsychopharmacology* **45**, 1215–1222 (2020). 32032968 10.1038/s41386-020-0630-2 PMC7235023

[28] Mayasi, Y. et al. Atrial fibrillation is associated with anterior predominant white matter lesions in patients presenting with embolic stroke. *J. Neurol. Neurosurg. Psychiatry*. **89**, 6–13 (2018). 28554961 10.1136/jnnp-2016-315457 PMC5704976

[29] Tullberg, M. et al. White matter lesions impair frontal lobe function regardless of their location. *Neurology* **63**, 246–253 (2004). 15277616 10.1212/01.wnl.0000130530.55104.b5 PMC1893004

[30] Ihara, M. et al. Quantification of Myelin loss in frontal lobe white matter in vascular dementia, Alzheimer’s disease, and dementia with lewy bodies. *Acta Neuropathol. (Berl)*. **119**, 579–589 (2010). 20091409 10.1007/s00401-009-0635-8 PMC2849937

[31] Yau, P. L., Hempel, R., Tirsi, A. & Convit, A. Cerebral White matter and retinal arterial health in hypertension and type 2 diabetes mellitus. *Int. J. Hypertens.* **2013**, e329602 (2013). 10.1155/2013/329602 PMC3745833 23984047

[32] Payabvash, S. et al. Regional ischemic vulnerability of the brain to hypoperfusion. *Stroke* **42**, 1255–1260 (2011). 21493917 10.1161/STROKEAHA.110.600940 PMC3090217

[33] Kalaria, R. N. Cerebrovascular disease and mechanisms of cognitive impairment. *Stroke* **43**, 2526–2534 (2012). 22879100 10.1161/STROKEAHA.112.655803

[34] Hase, Y. et al. White matter capillaries in vascular and neurodegenerative dementias. *Acta Neuropathol. Commun.* **7**, 16 (2019). 30732655 10.1186/s40478-019-0666-x PMC6366070

[35] Yamori, Y., Horie, R., Sato, M. & Fukase, M. Hemodynamic derangement for the induction of cerebrovascular fat deposition in normotensive rats on a hypercholesterolemic diet. *Stroke* **4**, 385–389 (1976). 960150 10.1161/01.str.7.4.385

[36] Brown, W. R. & Thore, C. R. Review Cerebral microvascular pathology in ageing and neurodegeneration. *Neuropathol. Appl. Neurobiol.* **37**, 56–74 (2011). 20946471 10.1111/j.1365-2990.2010.01139.x PMC3020267

[37] Allen, I. V., McQuaid, S., Mirakhur, M. & Nevin, G. Pathological abnormalities in the normal-appearing white matter in multiple sclerosis. *Neurol. Sci.* **22**, 141–144 (2001). 11603615 10.1007/s100720170012

[38] Restrepo, J., Bernardin, L. & Hammeke, T. Neurocognitive decline in Alexander disease. *Clin. Neuropsychol.* **25**, 1266–1277 (2011). 21902566 10.1080/13854046.2011.604043

[39] Tolhurst, D. J. & Lewis, P. R. Effect of myelination on the conduction velocity of optic nerve fibres. *Ophthalmic Physiol. Opt.* **12**, 241–243 (1992). 1408181 10.1111/j.1475-1313.1992.tb00298.x

[40] Chopra, S. et al. More highly myelinated white matter tracts are associated with faster processing speed in healthy adults. *NeuroImage* **171**, 332–340 (2018). 29274747 10.1016/j.neuroimage.2017.12.069

[41] Lloyd-Jones, D. M. et al. Framingham risk score and prediction of lifetime risk for coronary heart disease. *Am. J. Cardiol.* **94**, 20–24 (2004). 15219502 10.1016/j.amjcard.2004.03.023

[42] Hippisley-Cox, J., Coupland, C., Robson, J. & Brindle, P. Derivation, validation, and evaluation of a new QRISK model to estimate lifetime risk of cardiovascular disease: cohort study using QResearch database. *BMJ* **341**, c6624 (2010). 21148212 10.1136/bmj.c6624 PMC2999889

[43] Mons, U., Schöttker, B., Müller, H., Kliegel, M. & Brenner, H. History of lifetime smoking, smoking cessation and cognitive function in the elderly population. *Eur. J. Epidemiol.* **28**, (2013). 10.1007/s10654-013-9840-9 23990211

[44] Fry, A. et al. Comparison of sociodemographic and health-related characteristics of UK biobank participants with those of the general population. *Am. J. Epidemiol.* **186**, 1026–1034 (2017). 28641372 10.1093/aje/kwx246 PMC5860371

[45] Alfaro-Almagro, F. et al. Image processing and quality control for the first 10,000 brain imaging datasets from UK biobank. *NeuroImage* **166**, 400–424 (2018). 29079522 10.1016/j.neuroimage.2017.10.034 PMC5770339

[46] Josse, J. & Husson, F. missMDA: A package for handling missing values in multivariate data analysis. *J. Stat. Softw.* **70**, 1–31 (2016).

[47] Rosseel, Y. lavaan: An R package for structural equation modeling. *J. Stat. Softw.* **48**, 1–36 (2012).

